# The trend in adoption of hearing aids following changes in provision policy in South Korea

**DOI:** 10.1038/s41598-022-17459-7

**Published:** 2022-08-04

**Authors:** Hayoung Byun, Eun Mi Kim, Inah Kim, Seung Hwan Lee, Jae Ho Chung

**Affiliations:** 1 Department of Otolaryngology-Head and Neck Surgery, College of Medicine, Hanyang University, 222-Wangshimni-ro, Seongdong-gu, Seoul, 133-792 Republic of Korea; 2 Department of Big Data Management, National Health Insurance Service, Wonju, Republic of Korea; 3 Department of Occupational and Environmental Medicine, Hanyang University, Seoul, Republic of Korea

**Keywords:** Diseases, Health care, Medical research

## Abstract

The Korean government started to cover part of the price of hearing aids ($200) for individuals with hearing disabilities in 1997, and the reimbursement for a hearing aid increased in 2005 ($300) and again in 2015 ($1000). The aim of this study was to evaluate the annual trend of newly-registered individuals with hearing disability according to the changes of the hearing aid provision scheme. Subjects with newly-registered hearing disabilities were assessed using Korean National Health Insurance Service (KNHIS) data from January 1, 2004, to December 31, 2018. A total of 271,742 individuals were newly registered during the index period. Records of hearing aid prescriptions and hearing aid subsidies were used to assess the adoption of hearing aids. This study also assessed the intervals between registration of hearing disability and the adoption of hearing aids, as well as the number of hearing aid subsidies provided. From 2004 to 2009 there was a slight increase in the number of individuals newly registered with hearing disabilities, and from 2011 to 2015, the number showed a tendency to decrease. Then, from 2015, the number of individuals with hearing disabilities increased abruptly, and the proportion of subjects receiving hearing aid subsidies also increased. Between 2004 and 2018, the time interval from hearing disability registration to hearing aid adoption showed a decreasing trend. We conclude that the annual number of individuals with newly-registered hearing disabilities is affected by the level of the hearing aid subsidy, and there is much unregistered or unaddressed hearing loss prior to the introduction of realistic hearing aid provision.

## Introduction

Hearing loss is one of the most common disabilities, and negatively affects multiple aspects of individuals’ lives due to problems with speech recognition and communication^1,2^. Auditory deprivation can impair quality of life throughout life. Hearing loss impedes language development in childhood and contributes to dementia risk and cognitive decline in old age^3–5^. For these reasons, early diagnosis and proper hearing rehabilitation are important, and the role of hearing aids has been emphasized^6,7^.

However, the substantial out-of-pocket costs for hearing aids can be a major barrier to hearing rehabilitation^8^. A recent US community survey demonstrated that the average bundled cost of a hearing aid was $2500, and three-quarters of Americans with hearing loss could not afford a hearing aid^8^. The widespread lack of insurance cover for hearing aids could be one of the reasons for the poor uptake of hearing aids^9^.

All Koreans have been covered by medical insurance since 1989 with a single insurer named the National Health Insurance System (NHIS) of Korea, which provides partial subsidies for hearing aids to individuals registered as “hearing disabled” after comprehensive hearing evaluation. The hearing aid subsidy program started in 1997, with partial reimbursement (about $200) of the cost of a hearing aid, and this increased to $300 in 2005. In 2015, the hearing aid subsidy was increased to about $1,000, which is the price of a single entry-level hearing aid.

The present study aimed to identify trends in the annual number of newly-registered individuals with hearing disability, and of hearing aid use, according to changes in hearing aid provision.

## Methods

### Study population

National claims data related to insurance reimbursement provide information on medical institutions; personal information including hearing disability; disease classification according to the10th version of the International Classification of Diseases and Related Health Problems (ICD-10); medical history (medical tests, procedures, operations, and prescriptions); and medical costs. We examined National Health Information Database data (NHIS-2022-1-269) from January 2004 to 2018. Individuals who have hearing loss can be registered as hearing disabled for social and financial assistance including subsidies for hearing aids and discounts or exemptions from telecommunication and public transport charges, as well as for tax reductions. The present study assessed individuals with hearing disabilities and a history of hearing aid subsidies based on the NHIS database.

### Study design and subject selection

**Figure 1:**
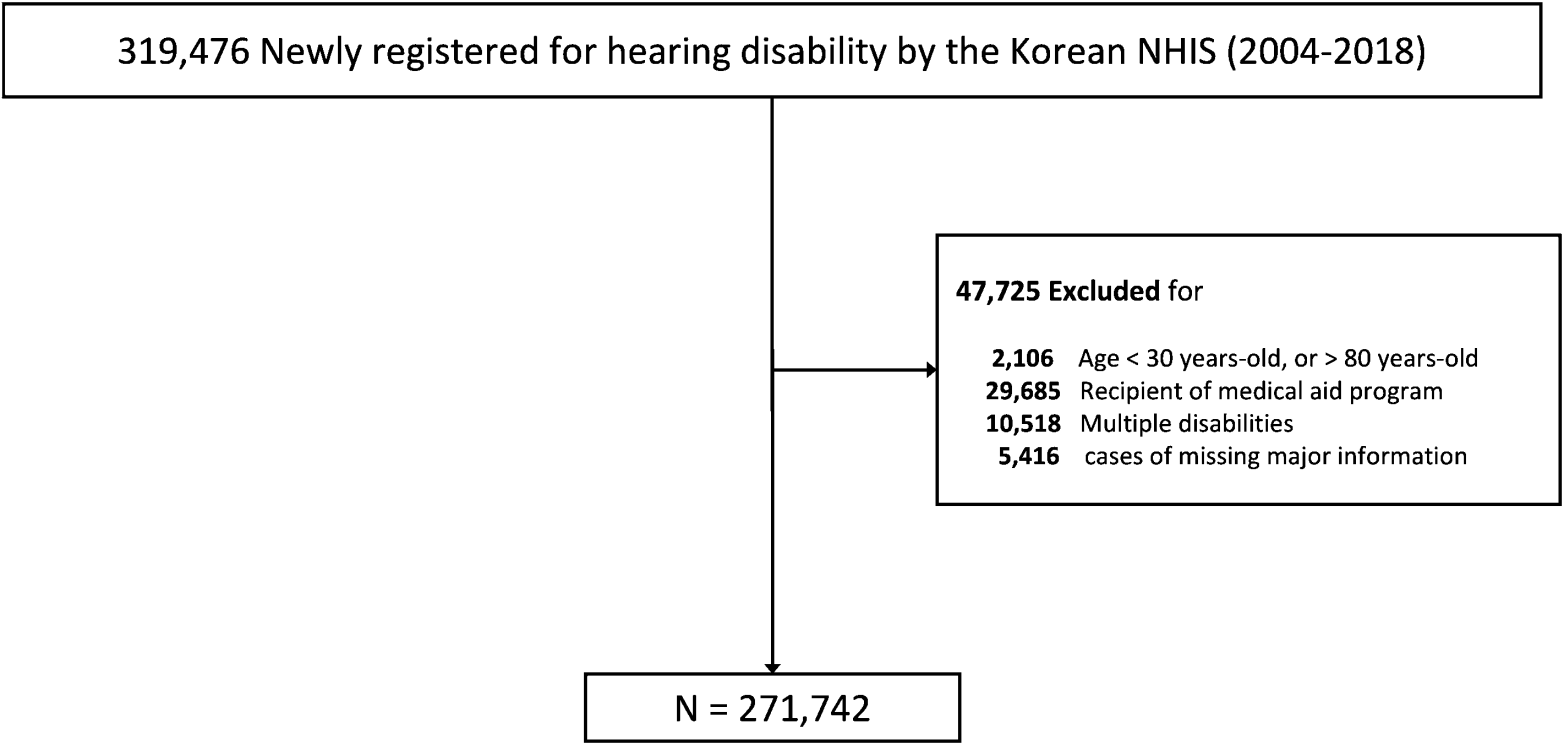
Flow diagram of the study. *NHIS* National health insurance system.

Medical claims data regarding hearing disability from January 2004 to December 2018 were analyzed retrospectively. Adult individuals 30–80 years old who were newly-registered with hearing disability during the index period were identified and evaluated. Recipients of the medical aid program for the extremely-low income group, individuals with multiple disabilities, and individuals with missing data were excluded from the analysis (Fig. 1).

### Hearing levels of individuals with hearing disabilities

Individuals with hearing loss could be registered as hearing disabled by undergoing three pure tone and speech audiometry tests, and a relevant audiometry brain stem response test to confirm fixed hearing loss. Hearing levels were calculated based on average pure tone thresholds at four frequencies using the following formula: (0.5 kHz + 1 kHz + 1 kHz + 2 kHz + 2 kHz + 4 kHz)/6. Based on these audiometric tests, an otolaryngology specialist would issue a medical certificate for hearing loss. The test results and medical certificates were reviewed by two otology specialists affiliated with the Korean government who would confirm the disability registration based on the audiometric threshold.

Hearing disability was classified into 5 levels: Grade 2 (better ear ≥ 90 dB HL), Grade 3 (better ear ≥ 80 dB HL), Grade 4 (better ear ≥ 70 dB HL), Grade 5 (better ear ≥ 60 dB HL), and Grade 6 (worse ear ≥ 80 dB HL and better ear HL ≥ 40 dB HL). In the current study, subjects with Grades 2 and 3 were classified as ‘profound hearing loss’ and those with grades 4, 5, and 6 as ‘moderate to severe hearing impairment’—terms closely resembling the World Health Organization grades of hearing impairment^10^.

### Hearing aid subsidies for individuals with hearing disabilities

The Korean government has been providing hearing aid payments to individuals with hearing disability once every 5 years since 1997; the amount was changed from about $200 per device to about $300 in 2005, and to $1000 per device in 2015. With a hearing aid prescription issued by otolaryngologists, individuals with hearing disability could purchase their hearing aids from distributors of their choice. Claims for hearing aids are paid to the sellers after an otolaryngologist checks the adopted device by direct inspection and aided audiometry, and issues a confirmation document.

### Hearing aid-related parameters

Demographic information on age, gender, household income and dwelling place was assessed. Use of a hearing aid was defined as individuals with a hearing aid prescription and history of hearing aid subsidy during the index period. Time intervals between hearing disability registration and hearing aid subsidy were also assessed.

### Statistical analysis

Data were analyzed using SAS Enterprise Guide software version 7.1 (SAS Institute, Inc., Cary, NC). The demographic characteristics of the study population were summarized as percentages for categorical variables, and means and standard deviations for continuous variables. The chi-square test was used to compare categorical variables between hearing aid users and non-users. The Exact Poisson Method was used to compare frequencies of newly- registered hearing disabled and hearing aid adoption rates. The annual trend of hearing aid subsidy during the index period was examined using data for the entire population of South Korea.

### Ethics

This investigation was approved by the local ethics review board (Hanyang University Institutional Review Board, HYUIRB 202101–013) and performed in accordance with the Declaration of Helsinki and good clinical practice guidelines. The Institutional Review Board waived written informed consent for this study as the KNHIS dataset consists of de-identified secondary data available for research purposes.

## Results

### Demographics of the study population

From 2004 to 2018, the total number of newly-registered adults with hearing disability was 271,741, of which 52.9% were males and 78.8% were over 65 years of age. Of these individuals 84.9% had moderate to severe hearing loss and only 15.1% had profound hearing loss.

During the index period, 190,401 (70.1%) individuals received one or more hearing aid payments while the other 81,341 (20.9%) had not purchased a hearing aid by the end of the index period in 2018 (Table 1). In addition, a higher proportion of the individuals in the hearing aid subsidy group were in the highest income quartile than in the no-hearing aid subsidy group (P < 0.001).

**Table 1:** Demographics of the study population.

N	Total		No hearing aid		Hearing aid		P*
	271,741	%	81,341	%	190,416	%	
**Gender**							
Male	146,465	53.9%	45,803	56.3%	100,662	52.9%	< .001
Female	125,277	46.1%	35,523	46.7%	89,754	47.1%	
**Age**							
≥ 30, < 65	66,325	24.41%	26,138	32.1%	40.187	21.1%	< .001
≥ 65	205,417	75.59%	55,188	67.9%	150.229	78.9%	
**Income (quartiles)**							
Lowest	41,417	15.24%	13,914	17.1%	27,503	14.4%	< .001
Lower mid	54,689	20.13%	18,127	22.3%	36.562	19.2%	
Upper mid	64,050	23.57%	18,862	23.2%	45.188	23.7%	
Highest	111,586	41.06%	30,423	37.4%	81.163	42.6%	
**Urbanized level**							
Metropolis	109,305	40.22%	33,428	41.1%	75,877	39.9%	< .001
Urban	104,163	38.33%	31,446	38.7%	72,717	38.1%	
Rural area	58,274	21.44%	16,452	20.2%	41,822	22.0%	
**Severity of hearing loss**							
Moderate to severe	228,889	84.23%	67,186	82.6%	161,703	84.9%	< .001
Profound	42,853	15.77%	14,140	17.4%	28,713	15.1%	
**Hearing aid subsidy (~ 2018)**							
No	81,341	29.93%					
Yes	190,401	70.07%					

*Chi-square test.

### Changes in numbers of newly registered hearing disabled individuals between 2004 and 2018

**Figure 2:**
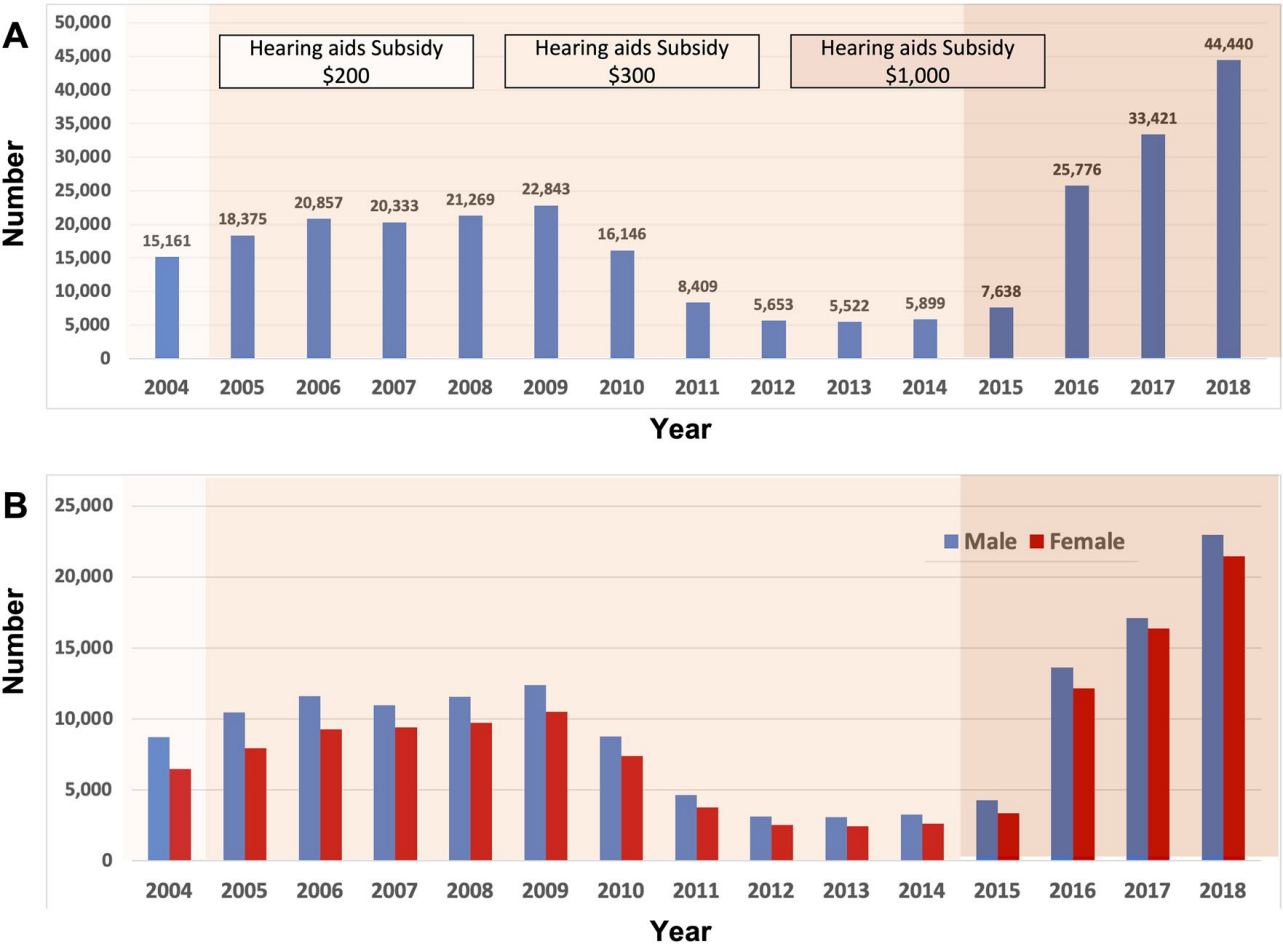
Numbers and gender distribution of newly-registered hearing disabled in South Korea from 2004 to 2018. (**A**) Numbers of individuals with newly registered hearing disability. (**B**) Gender distribution of hearing disabled individuals.

Table 2 and Fig. 2A show that the annual trend of newly-registered hearing disabled increased gradually from 2004 to 2009, decreased gradually from 2010 to 2014 and then increased abruptly from 2015 to 2018. The frequency of newly-registered hearing disabled in South Korea also increased up to 2009, decreased from 2010 to 2015 and increased from 2016 to 2018. Table 3 shows that the frequency of newly-registered hearing disabled was significantly higher in the period 2015–2018 than in the period 2005–2014. In addition, males predominated throughout the index period (Fig. 2B).

**Table 2:** Numbers of newly-registered hearing disabled according to disability grade in South Korea from 2004 to 2018.

Year	2004	2005	2006	2007	2008	2009	2010	2011	2012	2013	2014	2015	2016	2017	2018
Grade 2	1853	2159	1977	1733	1415	1325	464	326	255	275	268	252	452	363	309
Grade 3	3211	3699	3977	3705	3514	3556	818	609	501	458	466	594	1550	1493	1276
Grade 4	3537	4335	5372	5341	5905	6556	5344	2531	1428	1420	1463	2033	7069	8146	8864
Grade 5	4022	5172	6432	6764	7441	8271	6863	3532	2447	2440	2665	3516	13,239	19,101	28,451
Grade 6	2538	3010	3099	2790	2994	3135	2657	1411	1022	929	1037	1243	3466	4318	5540
Total	15,161	18,375	20,857	20,333	21,269	22,843	16,146	8,409	5,653	5,522	5,899	7,638	25,776	33,421	44,440
Population^a^	27,382,568	27,927,369	28,444,854	28,962,959	29,471,151	29,992,927	30,539,000	31,079,171	31,606,338	32,073,684	32,458,844	32,775,372	33,049,428	33,298,279	33,540,715
ratio (^0^/_000_)	5.5	6.6	7.3	7.0	7.2	7.6	5.3	2.7	1.8	1.7	1.8	2.3	7.8	10.0	13.2

Disability grade 2; better ear ≥ 90 dB HL, Grade 3; better ear ≥ 80 dB HL, Grade 4; better ear ≥ 70 dB HL, Grade 5; better ear ≥ 60 dB HL, and Grade 6; worse ear ≥ 80 dB HL and better ear HL ≥ 40 dB HL.

^a^Size of the eligible population from Census Data, Ratio (^0^/_000_); Per million, number of hearing disabled divided by total eligible population.

**Table 3:** Number of newly registered hearing disabled, and hearing aid adoption rate, according to size of hearing aid subsidy.

Parameter	Year		
	2005–2014	2015–2018	P-value
Amount of hearing aid subsidy	$300	$1,000	
Population (mean, n)^a^	30,255,630	33,165,979	
Newly-registered hearing disabled (mean, n)	14,530	27,818	
Proportion of newly-registered hearing disabled (^0^/_000_)	4.80	8.38	< 0.001
Hearing aid subsidy rate among the hearing disabled (%)	67.0%	75.6%	< 0.001

^a^Size of the Korean population from Census Data, ^0^/_000_; per million.

**Figure 3:**
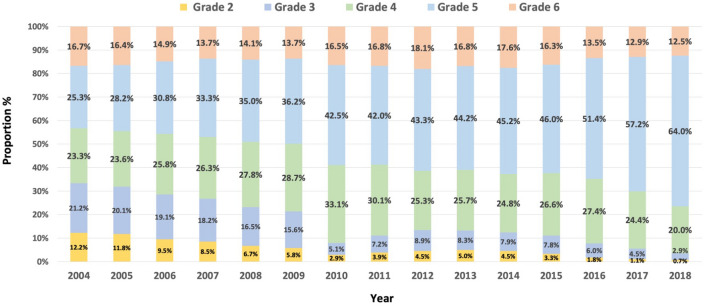
Distribution of grades of disability among newly-registered hearing disabled individuals in South Korea from 2004 to 2018. Disability grade 2; better ear ≥ 90 dB HL, Grade 3; better ear ≥ 80 dB HL, Grade 4; better ear ≥ 70 dB HL, Grade 5; better ear ≥ 60 dB HL, and Grade 6; worse ear ≥ 80 dB HL and better ear HL ≥ 40 dB HL.

Figure 3 illustrates the annual distribution of hearing disability grades throughout the index period. The proportion of individuals with profound hearing loss (Grade 2 or Grade 3) decreased, while the proportion with moderate to severe hearing loss (Grade 4, 5, and 6) increased.

### Hearing aid subsidy status in the hearing disabled population

The overall proportion of individuals with newly-registered hearing disabilities who received hearing aid subsidies was 70.07%. The mean hearing aid subsidy rate between 2015 and 2018 was significantly higher than between 2005 and 2014 (Table 3).

**Figure 4:**
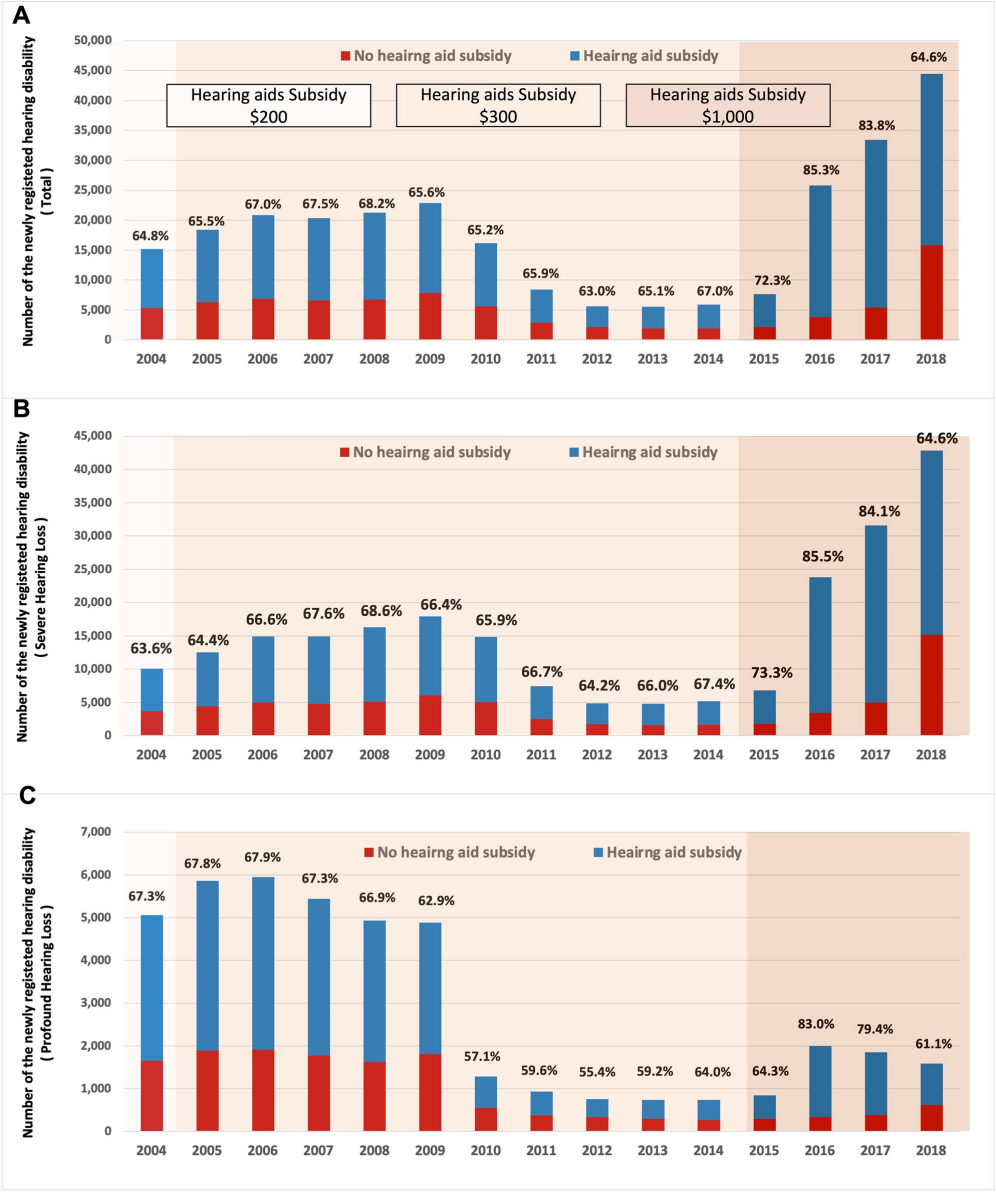
Total numbers of individuals with various grades of hearing loss, and proportion of individuals with hearing loss who received hearing aid subsidies by year. (**A**) Individuals with hearing disability. (**B**) Individuals with severe hearing loss. (**C**) Individuals with profound hearing loss.

The annual proportion of registered subjects who received hearing aid subsidies during the index period is shown in Fig. 4A. The proportion of individuals with newly-registered hearing disabilities who received hearing aid subsidies ranged from 63.0% to 67.5% from 2004 to 2014, and increased abruptly to 85.3% in 2016. Figure 4B,C show the annual trends in the proportion of individuals with newly-registered hearing disabilities who received hearing aid subsidies according to hearing level, and as a proportion of the total.

**Figure 5:**
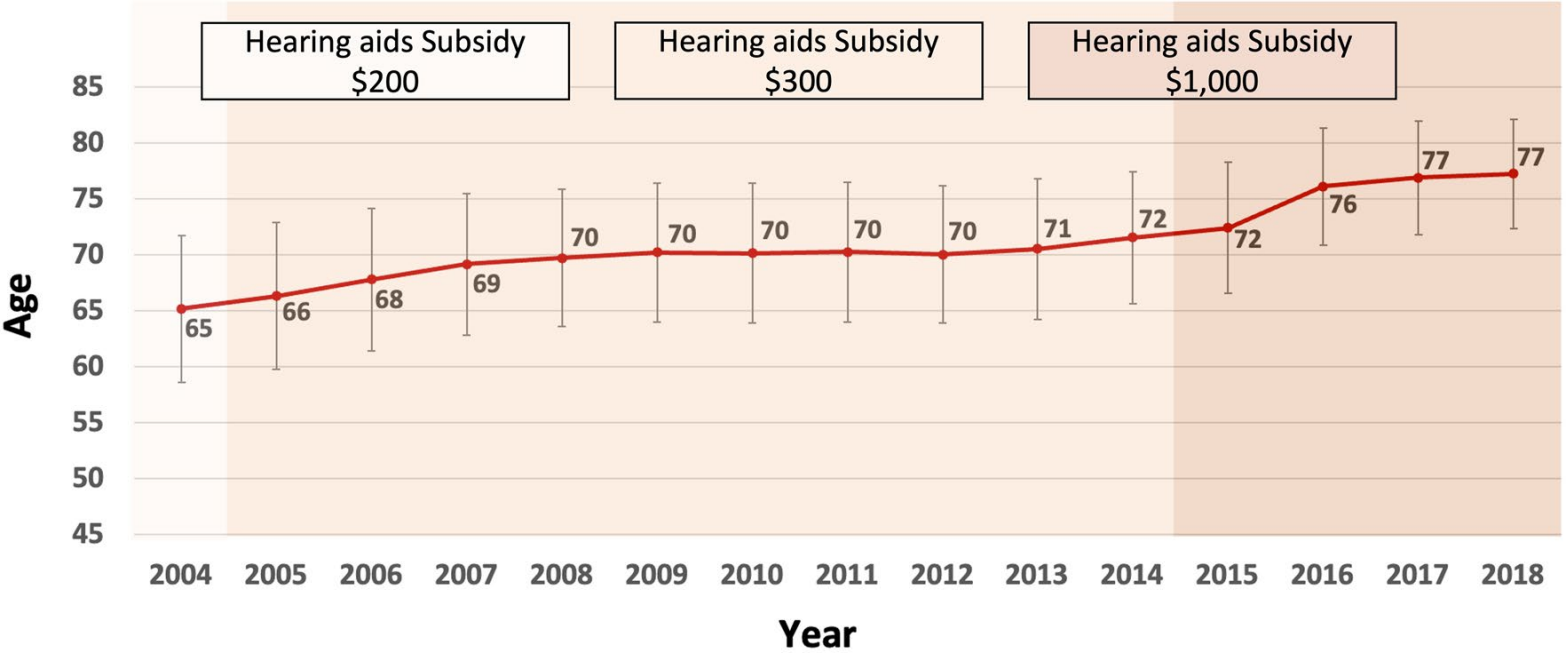
Mean ages of newly registered individuals with hearing disability between 2004 and 2018.

**Figure 6:**
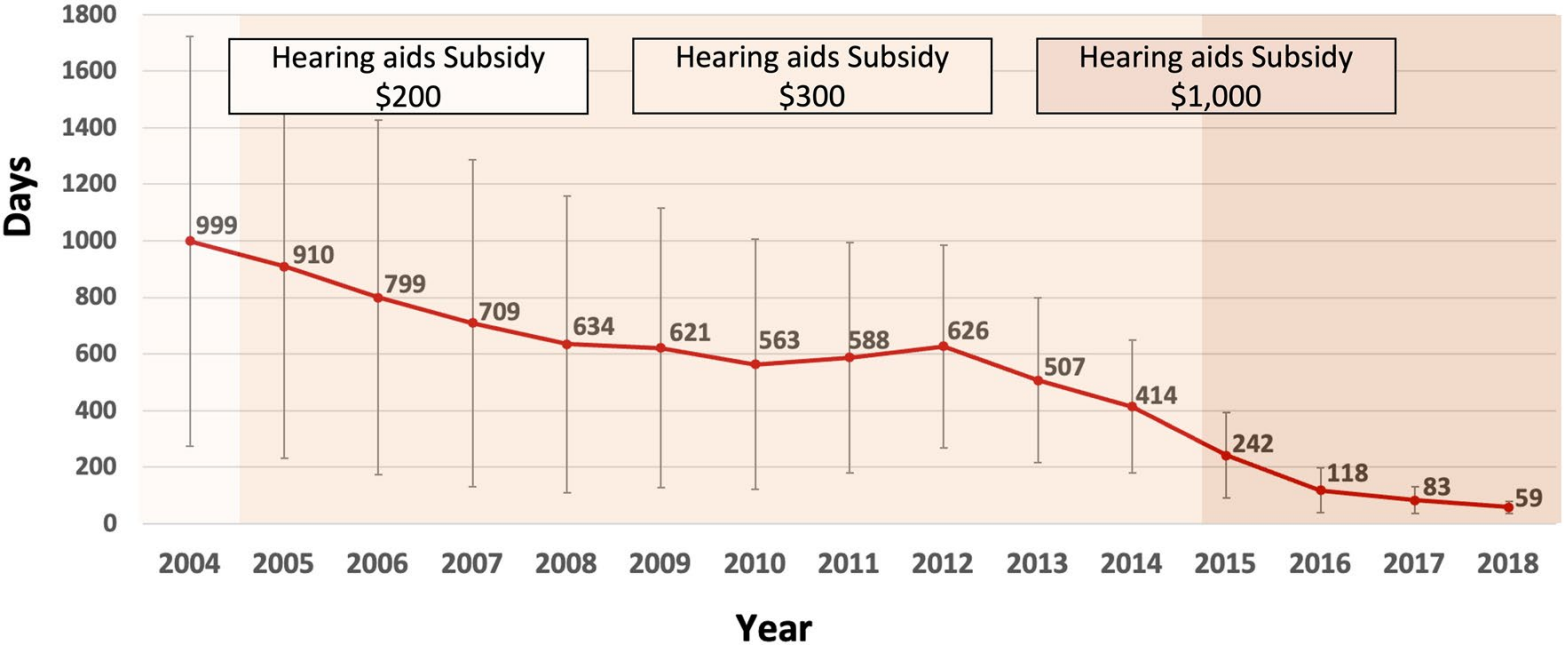
Mean time interval between registration of hearing disability and payment of hearing aid subsidies.

Interestingly, the mean age at the time of hearing disability registration increased gradually (Fig. 5). Also, the mean interval (days) between disability registration and payment of the hearing aid subsidy decreased gradually throughout the observation period (Fig. 6).

## Discussion

This study used nationwide insurance claims data to assess the effect of changes in the hearing aid provision scheme on annual trends in numbers of newly-registered hearing disabled and numbers of hearing aid subsidies paid. It found that the annual number of newly registered hearing disabled increased slightly and then decreased during periods of insufficient hearing aid subsidy (from 2004 to 2014) and rose dramatically in response to a substantial increase in the hearing aid subsidy (from 2015). In addition, the proportion of individuals with newly- registered hearing disabilities who received hearing aid subsidies increased to 85.3% in response to the large increase in hearing aid subsidy in 2015.

To our knowledge, this is the first official study to assess behavior regarding hearing aid adoption using a nationwide population database. The results indicate that a realistic hearing aid provision policy can facilitate rehabilitation behavior in individuals with hearing disability.

Hearing loss is the most common chronic condition in older individuals. The number of individuals worldwide with hearing loss is projected to reach 2.45 billion by 2050, a 56.1% increase from 2019^11^. Unaddressed hearing loss and inadequate hearing rehabilitation reduce quality of life in multiple ways, especially in terms of cognitive function^11^, and recognition of the social burden of hearing loss has grown. To alleviate the ill effects associated with hearing loss and its sequalae, hearing aid provision in an audiology clinic is the customary management procedure^12^. Despite the high prevalence of hearing impairment in old age, only a limited proportion of the hearing loss population who could potentially benefit from a hearing aid report current use of a hearing aid^13,14^. In the United States, approximately three-quarters of individuals with hearing loss cannot afford a hearing aid^8^. A Korean population study showed that only 17.4% of individuals with bilateral moderate to profound hearing loss purchased a hearing aid, and only about 73% of those individuals used the hearing aid regularly^14^. The importance of the diagnosis and management of hearing loss tends to be underestimated, especially in developing countries^11^.

The mean cost of a pair of fitted hearing aids ranged from $2,200 to $7,000 in 2014, and consumer reports show that the average price for a pair of hearing aids was $4,860 in 2021^15,16^. The hearing aid market is controlled by a limited number of companies, and the price of hearing aids is rather high and considered a first barrier to hearing aid adoption^15,17^. In other words, insurance coverage is a significant driver of hearing aid adoption^18^. According to data on welfare provisions for persons with disabilities, the South Korean government has been subsidizing a portion of the cost of 5-yearly purchase of hearing aids since 1997. The amount of the hearing aid subsidy increased from 250,000 won (about $200) to 340,000 won (about $300) in 2005 and was then tripled to 1,130,000 won (about $ 1,000) at the end of 2015.

A previous large study in South Korea examined the 10-year trend in the number of individuals who registered with profound hearing disability from 2006 to 2015^19^. It demonstrated that the trend of hearing loss showed a gradual decrease from 2010 to 2015^19^. In the present study, consonant with that report, the number of newly-registered individuals with hearing disability decreased from 2009 to 2013 (Fig. 2A). The decline in the number of newly-registered hearing disabled might be explained by supposing that most of those motivated by the second subsidy level ($300) may well have completed registration within the first 5 years.

However, we found that the number of newly-registered hearing disabled increased abruptly from 2015 at the same time as the level of the hearing aid subsidy increased. This finding suggests that that the price of hearing aids has a major effect on hearing aid adoption in South Korea. Another interesting point is that the previous study may have underestimated the actual number of individuals with hearing loss, since there are likely to have been many unregistered individuals with hearing loss before a more substantial hearing aid subsidy was introduced^19^. To be registered as hearing disabled in Korea, three pure tone audiometry tests and an auditory brainstem response test must be completed, and the cost of these auditory tests is about $250 ~ $300. Given that the cost of assessment approximated the hearing aid subsidy prior to 2015, there was little incentive for individuals with hearing loss to register as hearing disabled. Moreover, the actual value of the hearing aid subsidy would have decreased continuously during 2004–2014 if inflation was taken int account.

The present study also examined the annual trend of severity among newly registered hearing disabled from 2004 to 2018. The frequency of hearing disability grades 2 and 3, indicating profound hearing loss, among the newly-registered hearing disabled gradually decreased, while the proportion of individuals with severe hearing loss (disability grades 4 and 5) increased (Fig. 3). The mean age of newly registered individuals with hearing disability gradually increased from 2004 to 2015 and increased significantly thereafter (Fig. 5), implying that many older individuals with hearing loss had not registered before 2015. In addition, the mean time from disability registration to hearing aid adoption decreased greatly (Fig. 6), which suggests that the main purpose of disability registration might usefully be changed to the adoption of a hearing aid rather than to receipt of other social benefits for hearing disability.

This study demonstrated that reducing the cost to consumers/patients for hearing aids by expanding the hearing aid subsidy increased uptake in individuals with hearing loss who could benefit from a hearing aid. We identified an immediate increase in uptake of hearing aids following the expansion of hearing aid subsidies (Fig. 4). However, there are many other kinds of barriers to hearing aid adoption besides the price issue^20^. In Iceland, the national health insurance scheme fully covers hearing aid purchase every 4 years, but only 11% of those with hearing loss use hearing aids^21^. This low hearing aid adoption rate can be explained by the complexity of the factors underlying hearing aid use. Hearing aid adoption is influenced by an intricate interaction between personality, perceived social value, and social stigma^20,22^.

Several studies have attempted to quantify the financial results of hearing loss^23^. A retrospective cohort study in the United States found that the benefit of hearing aid adoption in individuals self-reporting hearing loss was identified as reducing the probability of emergency room visits and hospitalizations, and decreased Medicare spending^17^. However, use of a hearing aid led to increased office visits, and more total health care spending and out-of-pocket costs^17^. Another study proposed that the provision of hearing aids eventually adds value to the health care system and provides net savings to the Medicare program^24^. After 2015, individuals with severe to profound hearing loss could receive a hearing aid subsidy covering the cost of a single hearing aid under the South Korean national insurance system. It will be important to monitor whether expansion of the hearing aid provision scheme reduces the loss of quality of life associated with hearing disability in terms of access to the medical system and leads to a reduction of overall medical expenses, and this information should be reflected in any future changes to hearing aid provision. Lastly, hearing aid adoption does not necessarily imply regular use of the hearing aid^14,25^. Proper hearing aid fitting and checking, as well as counseling are important in establishing an appropriate and effective hearing rehabilitation environment^26^.

This study has the strength of using nationwide population data to investigate annual trends in numbers of newly-registered hearing disabled in response to changes in hearing aid provision. It successfully identified changes in hearing aid adoption according to the level of financial support. The study also has limitations, mostly related to the characteristics of the claims data, since they do not include physical examination data such as tympanic membrane status and exact hearing level. Also, since the national hearing disability registration scheme only includes individuals with severe to profound hearing loss, the present study could not assess the use of hearing aids by individuals with mild to moderate hearing loss. These limitations could be overcome in future by including additional large sources of medical data.

In conclusion, expansion of the Korean hearing aid provision scheme resulted in a dramatic increase in hearing disability registration and hearing aid adoption, indicating that there was much unregistered or unaddressed hearing loss prior to the introduction of realistic hearing aid provision.

## Data Availability

The KNHIS database was used with permission. The data that support the findings of the study are available from the corresponding author upon reasonable request.
